# Successful treatment of amniotic fluid embolism complicated by disseminated intravascular coagulation with rivaroxaban: A case report

**DOI:** 10.1097/MD.0000000000018951

**Published:** 2020-01-24

**Authors:** Hai-Di Wu, Zi-Kai Song, Hong-Yan Cao, Xiao-Yan Xu, Ming-Long Tang, Shuo Yang, Yang Liu, Ling Qin

**Affiliations:** Department of Cardiology, First Hospital of Jilin University, Changchun, 130031, China.

**Keywords:** amniotic fluid embolism, disseminated intravascular coagulation, novel oral anticoagulant, rivaroxaban

## Abstract

**Rationale:**

An amniotic fluid embolism (AFE) is a rare, lethal syndrome that is commonly associated with disseminated intravascular coagulation (DIC). Anticoagulation therapy is the most important strategy to inhibit excessive activation of the coagulation cascade in patients with AFE and DIC. At present, treatment of AFE with rivaroxaban has not been reported.

**Patient concerns:**

We report a 37-year-old woman (gravida 2, para 1) at 39 weeks’ gestation with irregular contractions of the uterus was admitted to the obstetrical department. Ten minutes after the spontaneous rupture of the membranes, the patient complained of dyspnea and dysphoria and exhibited cyanosis of her lips. The patient's blood pressure decreased and heart rate increased rapidly, and 2100 mL of unclotted blood flowed from her vagina within 1 hour. Her platelet count dropped to 21 × 10^−9^/L, and the results from routine coagulation tests, and D-dimer and fibrin degradation product tests were obviously abnormal.

**Diagnoses:**

According to the current research consensus, AFE with DIC should be considered immediately when sudden cardiovascular collapse occurs around the time of labor and delivery, followed by the development of coagulopathy and hemorrhage.

**Interventions:**

In addition, the variety of supportive treatments, rivaroxaban was used in anticoagulant therapy.

**Outcomes:**

At follow-up 30 and 60 days, there were no complaints of discomfort or abnormal laboratory assays. The patient recovered completely.

**Lessons:**

: This case highlights that rivaroxaban, as a direct inhibitor of activated factor Xa, demonstrates a good therapeutic efficacy for treating AFE with DIC.

## 1 Introduction

Amniotic fluid embolism (AFE) is an acute, maternal disease with extremely low incidence but extremely high mortality.^[1,2]^ The amniotic fluid represents the environment necessary for the fetus and contains not only fetal components, such as squames from the skin, mucin from the gut, lanugo hairs, and meconium, but also tissue factor (TF), tissue factor pathway inhibitor (TFPI), and thromboplastin-like materials that can induce and lead to a high pro-coagulant situation.^[3,4]^ Despite many new researches in this field, the precise etiology and pathogenesis of AFE remain unclear, and there is no gold standard diagnostic test or specific therapy for AFE. Currently, AFE remains a diagnosis of exclusion, dependent on bedside evaluation and judgment. Sudden onset of hypoxia, hypotension, and coagulopathy (including prolongation of coagulation times, hypofibrinogenemia and fibrinolytic activation, etc) and thrombocytopenia during labor and delivery in a short time is the hallmark of AFE diagnosis.^[5,6]^ At any stage of pregnancy, the mother can develop a strong allergic reaction to components of the amniotic fluid that enters the bloodstream, thereby activating proinflammatory mediators similar to that seen with the classic systemic inflammatory response syndrome.^[7]^ The nature of disseminated intravascular coagulation (DIC) in AFE is not completely understood. The accumulation of certain amniotic fluid substances can then activate various coagulation pathways, promoting the formation of systemic thrombosis mainly containing coagulation factors, triggering rapid and massive platelet aggregation that results in DIC in all organs of the body and vital organ failure.^[8,9]^ In order to inhibit the clotting activation, and reduce coagulation factor and platelet consumption, anticoagulation therapy is critical.

Fulminant intravascular accumulation of coagulation factors and platelets results in uncontrolled bleeding that is fatal, often within hours or days unless a timely diagnosis is made and appropriate treatment was provided. Unfortunately, the current recommendations for the management of AFE are limited due to the absence of controlled trials, and the available published reports offer no firm recommendations. Previous studies have only reported the use of heparin and warfarin to treat AFE and DIC.^[10,11]^ However, heparin may induce thrombocytopenia, treatment with heparin is difficult in patients with severe thrombocytopenia. We present a case of AFE and DIC who presented platelet count 21 × 10^–9^/L treated with oral rivaroxaban (15 mg twice daily), only 4 days later, the patient's clinical symptoms improved, the hyper-coagulable state was effectively controlled, platelet count returned to normal and the dosage was maintained for 3 weeks and then reduced to 20 mg once daily, total time was 3 months. All clinical symptoms and laboratory results had returned to normal, and good outcome was obtained.

## 2 Case presentation

A 37-year-old woman (gravida 2, para 1) at 39 weeks’ gestation who was experiencing irregular contractions of the uterus for 1 hour was admitted to the obstetrical department. She had given birth to a healthy female baby 5 years prior. At admission to the hospital, the patient's heart rate was70 beats/minute, blood pressure was 110/80 mm Hg, respiratory rate was 18 breaths/minute, and oxygen saturation was 98% on room air. An initial laboratory analysis demonstrated unremarkable results from platelet, hemoglobin, routine coagulation, and liver and renal function tests and normal D-dimer, and fibrin degradation products (Table 1). The fetal heart rate was 150 beats/minute. Within 4 hours of admission, the patient experienced a spontaneous rupture of the membranes. The amniotic fluid was clear and the cervix was dilated to 10 cm. After 10 minutes, the patient complained of dyspnea and dysphoria and exhibited cyanosis of her lips. Her blood pressure dropped to 98/60 mm Hg and heart rate increased to 122 beats/minute. The fetal heart rate dropped to 70 beats/minute. A live, 3150 g female infant was immediately delivered by forceps and subsequently transferred to the neonatal intensive care unit. Approximately 20 minutes until the expulsion of the placenta, completing the delivery, at this time, large amounts of unclotted blood flowed from her vagina; her blood loss was 2100 mL in 1 hour. Her blood pressure dropped to 40/20 mm Hg and her heart rate was 156 beats/minute. She was unconsciousness and exhibited pale lips and extreme dyspnea, and was thus transferred to the intensive care unit.

**Table 1:**
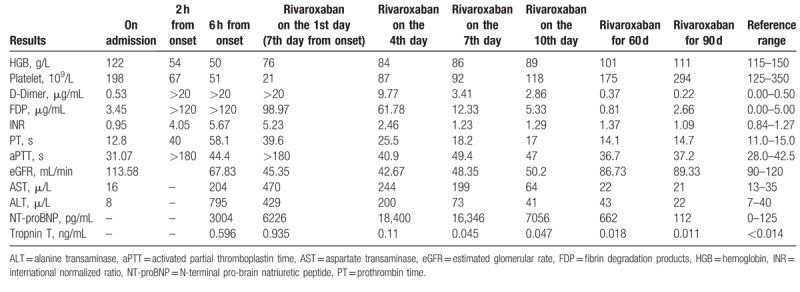
Laboratory results around the time in hospital and at follow-up.

The patient received intravenous transfusions of red blood cell suspensions, plasma from a fresh frozen supply, hemostatic drugs, large amounts of colloids, and crystalline solutions. She was placed on a ventilator, and hemofiltration and various support measures were carried out. Assay results revealed a marked decrease in platelets and hemoglobin, and the results of routine coagulation (including prothrombin time, international normalized ratio, and activated partial thromboplastin time), and D-dimer and fibrin degradation product tests were obviously abnormal (Table 1). AFE complicated by DIC was diagnosed. Her bleeding was brought under control, with a total blood loss of approximately 3000 mL. Her liver function and cardiac troponin-T, creatinine, and N-terminal pro-brain natriuretic peptide levels were elevated after 6 hours (Table 1). After 1 week of continuous blood transfusion, plasma input, blood filtration, mechanical ventilation, and a variety of supportive treatments the patient's condition was unstable, with low blood pressure, rapid heart rate, and 2 cardiac arrests. Although hemoglobin levels had significantly increased, platelets, routine coagulation D-dimer as well as fibrin degradation product test results continued to deteriorate (Table 1).

The above-described results indicate that coagulation factors and platelets were still activated and consumed excessively, resulting in serious damage to heart, liver, kidney, respiratory function, and coagulation system. Anti-coagulation therapy was imperative, and consultations with cardiology, intensive care, hematology, pneumology, and obstetric units were requested. The opinion was that heparin should not be used as an anticoagulant to prevent further heparin-induced platelet decline in patients with a marked reduction in platelet count. However, warfarin, a traditional anticoagulant, was not suitable for the patient's condition due to its work slowly and had to be bridged with heparin. After careful discussion and analysis, rivaroxaban is recommended for anticoagulation therapy. Rivaroxaban is direct factor Xa inhibitor, which is currently the most safe and effective anticoagulant treatment for VTE. Although rivaroxaban has no indication for the treatment of amniotic fluid embolism, we believe that rivaroxaban is the only treatment measure that may save the lives of patients and improve long-term prognosis under the current condition – the activation of the coagulation system after amniotic fluid embolism, leading to the further deterioration of the condition to DIC. Therefore, the consensus was to provide oral rivaroxaban, 15 mg twice daily. Four days after anticoagulation therapy with rivaroxaban, the patient's dyspnea improved, her blood pressure was 100/68 mm Hg and heart rates were 96 beats/minute, and the D-dimer levels dropped and platelet counts increased (Table 1). After 7 days, her situation improved significantly, the indexes gradually recovered, and mechanical ventilation was stopped (Table 1). After 10 days, the patient's routine coagulation and platelet count test results returned to almost normal (Table 1). She was maintained on 15 mg oral rivaroxaban twice a day for 3 weeks, and then the dose was reduced to 20 mg once a day. After discharge, the patient was followed up at 30 and 60 days. Her blood pressure and heart rate were normal, she reported no discomfort, and all previously abnormal laboratory results were normal (Table 1). The patient received oral rivaroxaban for a total of 3 months.

Ethical approval for our case report was waived by the First Hospital of Jilin University Ethical Board on the basis of their policy of reviewing all intervention and observational studies with the exception of case reports. The patient provided informed consent for the publication of her clinical data. The presented data are anonymized, and risk of identification is minimal.

## 3 Discussion

AFE remains one of the most devastating situations in obstetrics practice with high mortality of 20% to 60%.^[5]^ A recent study showed that the estimated incidence of AFE ranged from 0.8 to 1.8 per 100,000 maternities, and the proportion of women with AFE who died or had permanent neurological injury range from 30% to 41%.^[12]^ An analysis of the national registry found that 70% of cases of AFE occur during labor, 11% after a cesarean delivery, and 19% a vaginal delivery.^[13]^ For decades, studies on AFE have been progressing. The studies generally involved experimental animal models and clinical practice.^[12,14]^ Majority of studies showed that amniotic components were somehow forced into the maternal circulation during labor, delivery, or immediately postpartum, and amniotic fluid cellular debris was filtered by the pulmonary capillaries, resulting in obstruction of pulmonary arteries. Such obstruction leads to hypoxia, right ventricular heart failure, and death. Amniotic fluid was found to induce both platelet aggregation and the release of platelet factor III as well as activate factor X and the complement factors in vitro,^[13,14]^ leading to prolongation of coagulation times, hypofibrinogenemia, fibrinolytic activation and thrombocytopenia, and following by DIC, due to the large amount of coagulation factor and platelet depletion.^[5]^ DIC has been reported to occur in approximately 80% of patients with amniotic fluid embolism syndrome^[14]^ and may ultimately be the principle cause of death.

As we all know, pregnancy is a state of relative hyper-coagulability, with increased levels of fibrinogen, factors VIII, IX, and X, and von Willebrand factor. Although the pathophysiology of AFE and the precise mechanism of the pro-coagulant activity are still not well understood, the acute coagulopathic peripartum calamities may be triggered by TF, released from the placenta and amniotic fluid.^[11]^ High levels of TFPI in amniotic fluid during normal pregnancy may be associated with pro-coagulant activity.^[8]^ The coagulation factor activation and platelet consumption contribute to DIC, a syndrome that is also secondary to a variety of clinical conditions associated with high mortality and morbidity rates. Despite a rapid diagnosis and intensive critical management, patients often do not recover from the exacerbation conditions.

Risk factors of AFE are inconsistent and contradictory and no putative risk factor has been identified. These reported risk factors for AFE included situations in which the exchange of fluids between the maternal and fetal compartments is cesarean delivery, placenta previa, instrumental delivery, cervical trauma, and abruption, induction of labor.^[5,15]^ Other reported risk factors include advanced maternal age and parity, male fetus, eclampsia, polyhydramnios, and multiple gestations.^[5,16]^ Until now, there is no consistent consensus for AFE prevention. Clinicians should fully assess the risks and benefits of induced labor and cesarean section to minimize the risks of potentially fatal AFE. When AFE is suspected, an obstetrician and/or anaesthetist present at the time of the AFE event and use of intervention to correct coagulopathy may be important to improve maternal outcome. Initial high-quality supportive obstetric care is also associated with a better AFE prognosis. Future research should focus on early detection of the coagulation deficiencies and coagulopathy management strategies in AFE.^[14,15]^

Our case was a 37-year-old woman at 39 weeks’ gestation with irregular contractions who was admitted to the obstetrical department. Soon after the spontaneous rupture of the membranes, she complained of dyspnea and exhibited a drop-in blood pressure and increase in heart rate, which suggest activation of the coagulation cascade, resulting from the presence of amniotic fluid in her blood. The subsequent blood loss after the placental delivery exacerbated her low blood pressure and elevated heart rate, resulting in a state of unconsciousness. On the basis of her symptoms and test results, she was diagnosed with AFE complicated by DIC.

There are no established guidelines for diagnostic tests with demonstrated accuracy for AFE and no proven therapies. As a result, many of the therapeutic decisions made are controversial and lack validation. Although the prompt initiation of supportive care may decrease the risk of mortality, blockade of the coagulation cascade and restoration of anti-coagulant pathways are vital.^[3]^ There are several reports on the varied use of heparin or warfarin, with some effect, in some patients with AFE and DIC.^[10,11]^ A study reported that a woman with AEF successfully treated by veno-arterial extracorporeal membrane oxygenation.^[17]^ However, there is no consensus on how to choose the appropriate anti-coagulation agent. Our patient was presented with severe thrombocytopenia: platelet counts dropped from 198 × 10^–9^/L (reference range, 125–350 × 10^–9^/L) to 21 × 10^–9^/L. To avoid further an exacerbation of this with heparin (heparin induce thrombocytopenia), we chose to administer oral rivaroxaban, which successfully resolved the abnormal test indices and the patient's symptoms.

Rivaroxaban is a novel oral anti-coagulant agent and a selective, direct factor Xa inhibitor. It is used to prevent and treat venous thromboembolism,^[18]^ to prevent stroke or systemic embolism in atrial fibrillation,^[17]^ and results in better cardiovascular outcomes in patients with stable atherosclerotic vascular disease.^[19]^ It has a predictable anti-coagulant effect, eliminating the need for routine coagulation monitoring. Compared with vitamin K antagonists, it also has a better efficacy/safety ratio, fewer food and drug interactions, a more rapid onset of action, and reduced risk of fatal bleeding. On the basis of this and the positive results of large trials and current guidelines, rivaroxaban should be considered the first preferred anti-coagulation therapy for the majority of patients. With consideration of the renal dysfunction in the patient presented in this case report, computed tomography pulmonary angiography was not performed to confirm pulmonary embolism. However, AFE, like pulmonary embolisms, leads to secondary systemic thromboembolism, requiring prompt anti-coagulation. The patient was prescribed oral rivaroxaban treatment on the basis of EINSTEIN-PE trials and guidelines for pulmonary embolism therapy,^[18]^ resulting in a satisfactory outcome.

In summary, this is the first reported case for the successful treatment of AFE with rivaroxaban. Compared to that for traditional anticoagulants, the clinical application of rivaroxaban is short, but it has good efficacy in preventing and treating venous thromboembolism and preventing stroke or systemic embolism in AF. The case described here provides new treatment information for clinicians. Additional reports of treatment in similar cases will provide further support for such beneficial treatment of AFE with DIC.

## Acknowledgments

We thank all participants for their supports and participation.

## Author contributions

**Data curation:** Ming-Long Tang, Shuo Yang, Yang Liu.

**Project administration:** Xiao-Yan Xu.

**Visualization:** Hong-Yan Cao.

**Writing – original draft:** Hai-Di Wu, Zi-Kai Song.

**Writing – review & editing:** Ling Qin.
